# Association of Bosworth, Pilon, and Open Talus Fractures: A Very Unusual Ankle Trauma

**DOI:** 10.1155/2019/6316137

**Published:** 2019-02-10

**Authors:** Kevin Moerenhout, Georgios Gkagkalis, Rayan Baalbaki, Xavier Crevoisier

**Affiliations:** Service of Orthopaedics and Traumatology, Lausanne University Hospital, Rue du Bugnon 46, CH-1011 Lausanne, Switzerland

## Abstract

**Introduction:**

A Bosworth fracture-dislocation is a rare lesion resulting in a fixed dislocation of the distal fibula behind the posterior tibial tubercle. Only few cases have been reported showing an associated consequent fracture, namely, a pilon or a medial malleolus fracture.

**Case Report:**

We present a case report of a patient with an unusual combination of a Bosworth injury with a pilon fracture and an open multifragmentary talus fracture and our approach for open reduction and internal fixation. At one year postoperative, the patient developed an invalidating tibiotalar and subtalar arthrosis that eventually required an ankle-hindfoot arthrodesis. A Bosworth injury is an infrequent entity and is even rarer when associated with other fractures. Careful preoperative planning is necessary, as the combination of these fractures is a surgical challenge. Special care must be taken to preserve the neurovascular bundle.

**Discussion:**

The present case highlights a Bosworth injury involving a severity that has never been described before and suggests adding an eighth stage to the classification presented by Perry et al.

## 1. Introduction

A Bosworth fracture-dislocation is a rare lesion of the ankle resulting in a fixed dislocation of the lateral malleolus behind the posterior tibial tubercle. It was named after Dr. David Bosworth who reported about five cases in 1947 [1]. A variant of this lesion, with an avulsion of the distal tibia, has been described in some case reports. However, only few cases have been reported showing an associated consequent fracture, namely, a pilon fracture [2–4] or a medial malleolus fracture [5]. To the best of our knowledge, no publication has described an association of a Bosworth injury with a distal tibia fracture together with an open multifragmentary talus fracture of the ipsilateral ankle.

## 2. Case Presentation

**Figure 1:**
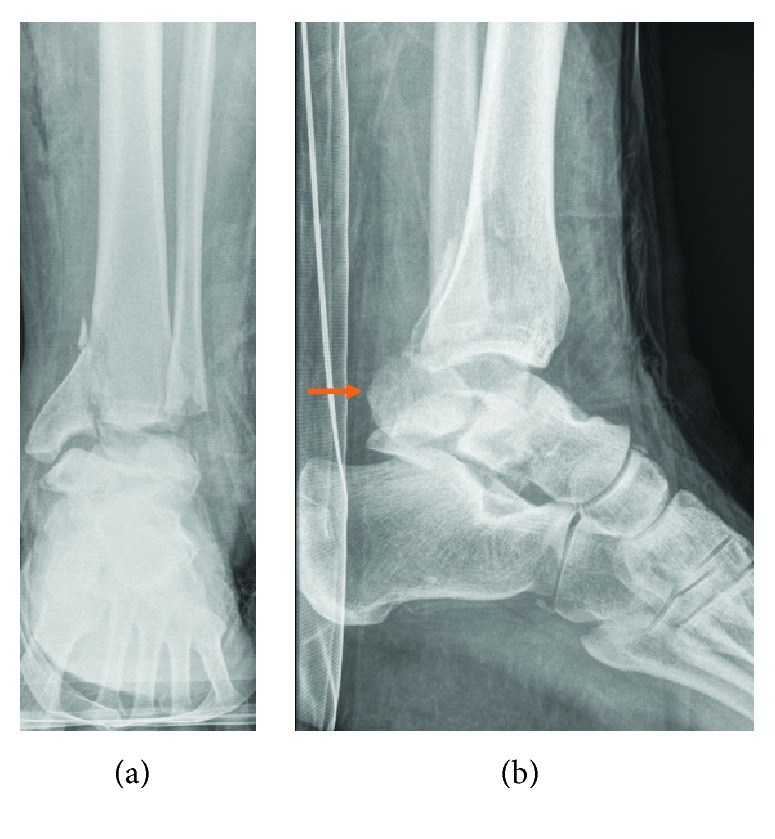
(a) Preoperative AP view of the left ankle: vertical shear fracture of medial distal tibia, horizontal lateral malleolus fracture, and talus fracture. (b) Preoperative lateral view of the left ankle: posterior dislocation of the distal fibula, talar neck fracture, and distal tibia fracture. The array shows the distal fibula dislocated posterior of the tibia and talus.

**Figure 2:**
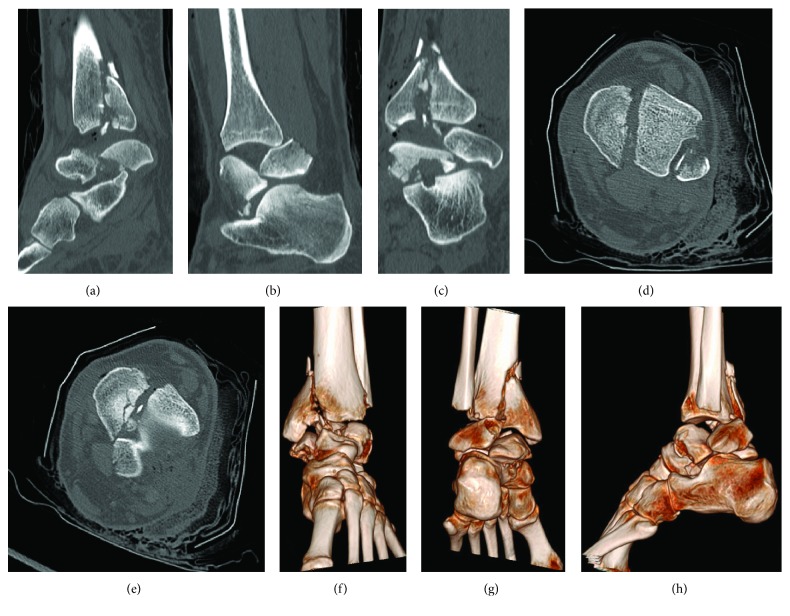
Preoperative CT scan of the left ankle with (a) the sagittal view of the pilon fracture and the split fracture of the talus, (b) distal fibula pushing the talus fragment anterior, (c) frontal view of the pilon fracture and the talus split fracture, (d) axial view with the fibula in the incisura, (e) fibula splitting the tibial plafond, and reconstruction of the left foot in (f) frontal anterior, (g) frontal posterior, and (h) sagittal lateral views.

A 44-year-old male constructor worker fell down from a 3-storey high building and presented to our Emergency Department. An open fracture of the left talus Gustilo type 3b was visible with an external submalleolar wound of 7 centimetres. Peripheral pulses were present, and the neurological status was intact. Plain film radiographs showed a posterior dislocated distal fibula fracture, a comminuted vertical shear fracture of the medial distal tibia and a talus fracture (Figure 1). In order to obtain a precise diagnosis and plan surgery, a computed tomography (CT) scan of the ankle was performed, showing a dislocated distal fibula in contact with the posterior medial part of the talus, a multifragmentary talus fracture with a sagittal split and a separation between the body and neck and an AO 43-B2 distal tibia fracture (Figure 2).

Considering an open fracture with an irreducible external malleolus, immediate surgery was performed. We used the open wound that extended from the proximal part of the external malleolus fracture to the cuboid bone to approach the fractures from the lateral side. First, the distal lateral malleolus was extricated as it was located between the distal tibia and posterior talus and fixed with two axial 2.2 Kirschner wires. The Chaput fragment was also stabilized with a single 1.2 Kirschner wire. These three wires were cut to the desired length, bent, and impacted into the bone. We then approached the medial aspect of the ankle, through an incision extending from 5 cm above the tip of the medial malleolus to the medial tuberosity of the navicular bone. Reduction and fixation of the talus with two K-wires and two partially threaded screws were accomplished. The quality of reduction was controlled on both medial and lateral approaches. Osteosynthesis of the pilon fracture was performed with a reconstruction plate. Intraoperative testing did not reveal syndesmotic instability.

**Figure 3:**
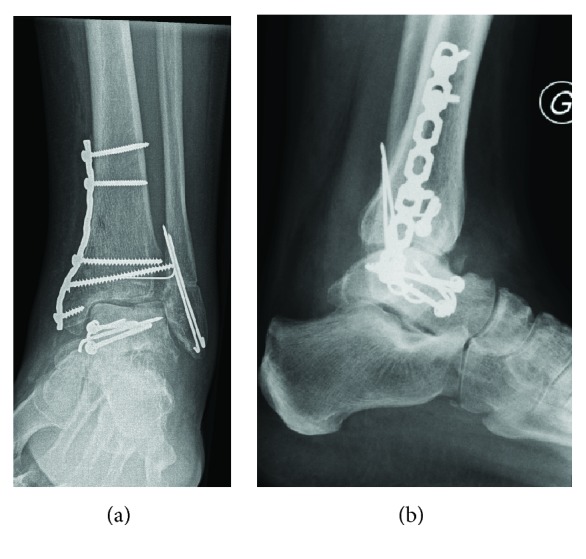
(a) AP and (b) lateral X-rays of the left ankle at 6 months, showing consolidated fractures and an anatomical reconstruction, with the absence of necrosis of the talus.

Postoperatively, a short leg cast was applied for a period of 12 weeks. Rehabilitation protocol consisted in 6 weeks of nonweight-bearing and a progressive weight-bearing starting from week 7. No acute complication was observed. At 6 months of follow-up, the fractures were consolidated. The X-rays showed an anatomical reconstruction, with no sign of talar necrosis (Figure 3). Passive flexion/extension of the ankle was limited to 20/0/0. The patient was able to walk for limited periods and distance only with adapted shoes and needed a daily pain medication intake. He was unable to regain his previous working status. Resuming sport activities was not possible.

**Figure 4:**
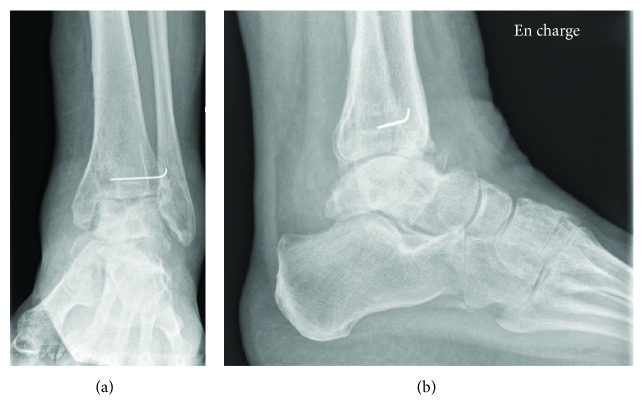
(a) AP and (b) lateral X-rays of the left ankle at 1 year, showing necrosis of the talus and pilon, with anteromedial articular conflict.

At one year postoperative, the patient developed an invalidating partial necrosis of the talus and pilon (Figure 4) that eventually required an ankle-hindfoot arthrodesis 14 months after the accident.

## 3. Discussion

Perry et al. classified the lesions involved in a Bosworth injury on a cadaveric study [6]. They described a seven-stage injury pattern, with stage one being the rupture of the anterior tibiofibular ligament. Stage two implicates the rupture of the posterior tibiofibular ligament. Stage three involves a rupture of the anteromedial part of the capsule. Stage four is a tear of the interosseous membrane. The fifth stage consists in a posterior entrapment of the fibula behind the tibia, followed by the fracture of the fibula as the sixth stage. The last stage can involve the medial malleolus or the deltoid ligament. This last stage has been described several times in the literature, but to the best of our knowledge, it has never been described in combination with a talus fracture.

Most cases of the Bosworth injury in the literature describe an injury classified as a Lauge-Hansen supination-external rotation (SER) fracture [7]. In our case, looking at the transverse fibula fracture associated with an AO 43B2 distal tibia fracture, we can assume that the lesions resulted from a supination-adduction movement combined with axial compression. It remains unclear, however, if the fracture of the talus was caused by the rotation mechanism or rather resulted from the axial impaction forces or even must be considered the combination of both.

In summary, in our case, even if we consider axial compression as a significant part of the mechanism of injury, we are convinced that the fracture pattern observed is only possible with rotational forces as the major component. Therefore, we decided to classify this complex lesion as a Bosworth injury. Given the fact that the combination of a stage seven Bosworth injury has never been described in combination with a talus fracture, we suggest adding the fracture pattern described in our case as stage eight to the Perry classification (Table 1).

Like most of the Bosworth fracture-dislocations described in the literature, treatment by closed reduction and casting was not possible. We took advantage of the open nature of the fracture on the lateral side of the hindfoot to reduce and fix the incarcerated distal fibula fracture. Special care was taken not to damage the posterior neurovascular bundle as the distal fibula was situated between the tibial nerve, the posterior tibial artery, and the flexor digitorum longus. Postoperative neurovascular assessment was normal. Posteromedial approach would have been a safer option to grab the distal fibula and would probably have permitted to reduce and stabilize the distal tibia too, but we would have been obliged to perform a supplementary anteromedial approach to fix the medial part of the talus.

The use of a reconstruction plate on the internal side of the distal medial tibia can be debated. In this case, although there was a tibia plafond fracture which should usually benefit from an optimal rigid fixation, the fracture behaved like a vertical shear medial malleolus-type fracture. For this reason, we considered the use of a reconstruction plate in an antiglide mode as a good option. Concerning the fixation of the lateral malleolus, a plate and screw construct would have been the optimal fixation. However, due to the fracture wound under the lateral malleolus, a further proximal dissection of the distal fibula was not undertaken, as it would have created supplementary cutaneous damage. Concerning the fixation of the talus, no fixation material was applied on the lateral side as it would have needed further distal dissection.

Intra-articular fracture management suggests aiming for anatomic reduction in order to optimize residual function and reduce the risk of developing posttraumatic osteoarthrosis, which is known to be associated with poor outcome [8–10]. However, Fournier et al. in a review of 115 cases did not find the functional outcome to be related to the quality of reduction [11]. Previous studies have also shown high rates of posttraumatic arthritis and osteonecrosis after talus neck fractures [12, 13]. In our case, the patient developed an early tibiotalar and subtalar arthrosis. Despite the poor outcome, surgical fracture management allowed at least an anatomic reduction with adequate ankle and hindfoot alignment, which then permitted an optimal position for the arthrodesis.

In conclusion, Bosworth fracture-dislocation is a rare entity and is even rarer if associated with other fractures like those of the talus or of the distal tibia. They remain a challenging fracture to treat because posttraumatic arthrosis is frequent and functional outcome is poor. Our case highlights a Bosworth injury involving a severity that has never been described before and suggests adding an eighth stage to the classification presented by Perry et al.

## Figures and Tables

**Table 1:** Different stages involved in Bosworth injury. Seven stages are proposed by Perry et al. on a cadaveric study. In italic, complementary modifications proposed by the authors.

Stages	Involved lesion in Bosworth injury
1	AITFL
2	PITFL
3	Anteromedial part of the capsule
4	Interosseous membrane
5	Posterior entrapment of the fibula behind the tibia
6	Fibula fracture
7	Associated medial malleolus fracture or the deltoid ligament torn *or pilon fracture*
*8*	*Associated talus fracture*

AITFL: anterior inferior tibiofibular ligament; PITFL: posterior inferior tibiofibular ligament.
